# Differences in Transcriptional Activation by the Two Allelic (L162V Polymorphic) Variants of PPAR*α* after Omega-3 Fatty Acids Treatment

**DOI:** 10.1155/2009/369602

**Published:** 2009-02-25

**Authors:** Iwona Rudkowska, Mélanie Verreault, Olivier Barbier, Marie-Claude Vohl

**Affiliations:** ^1^Lipid Research Center, CHUL Research Center, QC, Canada G1V 4G2; ^2^Department of Food Science and Nutrition, Laval University, QC, Canada G1V 0A6; ^3^Nutraceuticals and Functional Foods Institute (INAF), Laval University, QC, Canada G1V 0A6; ^4^Laboratory of Molecular Pharmacology, Oncology and Genomic Research Center, CHUL Research Center, QC, Canada G1V 4G2; ^5^Faculty of Pharmacy, Laval University, QC, Canada G1V 0A6

## Abstract

Omega-3 fatty acids (FAs) have the potential to regulate gene expression via the peroxisome proliferator-activated receptor *α* (PPAR*α*); therefore, genetic variations in this gene may impact its transcriptional activity on target genes. It is hypothesized that the transcriptional activity by wild-type L162-PPAR*α* is enhanced to a greater extent than the mutated variant (V162-PPAR*α*) in the presence of eicosapentaenoic acid (EPA), docosahexaenoic acid (DHA) or a mixture of EPA:DHA. To examine the functional difference of the two allelic variants on receptor activity, transient co-transfections were performed in human hepatoma HepG2 cells activated with EPA, DHA and EPA:DHA mixtures. Results indicate that the addition of EPA or DHA demonstrate potential to increase the transcriptional activity by PPAR*α* with respect to basal level in both variants. Yet, the EPA:DHA mixtures enhanced the transcriptional activity to a greater extent than individual FAs indicating possible additive effects of EPA and DHA. Additionally, the V162 allelic form of PPAR*α* demonstrated consistently lower transcriptional activation when incubated with EPA, DHA or EPA:DHA mixtures than, the wild-type variant. In conclusion, both allelic variants of the PPAR*α* L162V are activated by omega-3 FAs; however, the V162 allelic form displays a lower transcriptional activity than the wild-type variant.

## 1. Introduction

Higher intake of long-chain n-3 fatty acids (FAs) eicosapentaenoic acid (EPA) and docosahexaenoic acid (DHA) has been recommended to decrease plasma triglyceride (TG) levels. Conventionally, the mechanism of action after n-3 FAs intake focused on plasma membrane fluidity; however, recently the emphasis shifted to regulation of gene expression [1]. In particular, FAs and their derivatives are physiological ligands of peroxisome proliferator-activated receptor *α* (PPAR*α*). As such they activate the PPAR*α*-retinoid X receptor (RXR) heterodimer-dependent gene transcription by binding to the peroxisome proliferator response elements (PPRE) in the promoter region of target genes [2]. Target genes include lipoprotein lipase (LPL) involved in plasma TG clearance [2].

Several polymorphisms within the PPAR*α* gene and the encoded proteins have been identified including L162V and V227A, which are the most common PPAR*α* polymorphisms reported [3]. Of particular interest, the PPAR*α* L162V polymorphism has been associated with obesity indices and plasma lipid levels in numerous studies [4–8]. Additionally, Robitaille et al. in 2004 found that the interaction between the PPAR*α* L162V polymorphism and fat intake estimated from a food frequency questionnaire (FFQ) explains a significant percentage of the variance observed in waist girth in a sample of 260 French-Canadians [9]. Tai et al. in 2005 [10] also established that the effect of the PPAR*α* L162V polymorphism on plasma TG and apolipoprotein (apo)-CIII concentrations depends on the dietary polyunsaturated FA (PUFA), with a high intake triggering lower TG in carriers of the V162-PPAR*α* variant. Finally, Paradis et al. in 2002 [11] demonstrated that the interindividual variations in total cholesterol, apo A-I, and cholesterol concentrations in small low-density lipoprotein (LDL) particles observed after modification of the polyunsaturated/saturated FA ratio of the diet is partly attributable to the PPAR*α* L162V polymorphism. Clearly, both epidemiological and interventional studies demonstrate a relation between the PPAR*α* L162V polymorphisms, metabolic parameters, and FAs intake; yet, only two functional studies examined the receptor activity of the L162V polymorphic variants activated with synthetic agonists-fibrates [4, 12]. It was demonstrated that the effect of the L162V polymorphic variants on the transcriptional activation was associated with the concentration of the ligand to which it is exposed [12].

For that reason, the aim of this functional study was to determine whether the transcriptional activity by the wild-type variant, L162-PPAR*α*, is enhanced in the presence of natural PPAR*α* agonists-omega-3 FAs, mimicking the action of synthetic PPAR*α* agonists, comparatively to the variant, V162-PPAR*α*.

## 2. Laboratory Methods

### 2.1. Plasmid Construction

The wild-type L162-PPAR expression plasmid (pSG5-hPPAR*α* vector) was a kind gift from Pr. B. Staels (Unité INSERM 545, Institut Pasteur de Lille, France). The pSG5-mRXR*α* plasmid was described previously [13]. The V162-PPAR*α* expression plasmid was derived from the wild-type, through site-directed mutagenesis (QuickChange site-directed mutagenesis kit, Stratagene, La Jolla, Calif, USA) using the 5′-CGATTTCACAAGTGC**G**TTTCTGTCGGGATG-3′ oligonucleotide (the nucleotide in boldface type denotes the C→G change mutated base). Variant cDNAs were directly sequenced to confirm that no spurious base changes have been introduced during the procedure. As a response element representative of the vast variety of human PPREs, we choose the consensus artificial direct repeat (DR)1 sequence for analyzing the functional consequences of L162V variation on PPAR*α* activation by omega-3 FAs. Thus, a reporter plasmid (DR1x6-PPRE) was generated by cloning in front of the thymidine kinase promoter-driven luciferase reporter gene (TKpGL3 vector), six copies of the 5′-AAAACTAGGTCAAAGGTCACGG-3′ sequence where underlined nucleotides correspond to the direct repeat of the AGGTCA hexamer.

### 2.2. Transient Transfection and n-3 Fatty Acids Activation

Human hepatoma HepG2 cells were cultured in Dulbecco's Modified Eagle Medium (DMEM) supplemented by 10% fetal bovine serum (FBS), 1% of streptomycin penicillin, 1% of sodium pyruvate and 1% of glutamine. HepG2 cells were plated at a density of 75 × 10^3^ cells/well of 24-well plates which were then transfected using the ExGen reagent (Invitrogen, Burlington, Canada) with 50 ng of the DR1x6-PPRE reporter plasmid, 10 ng of the PPAR*α* (wild-type or mutant) and *RXR* expression plasmids and 30 ng of the pRL-NULL expression vector (Promega) for 6 hours 37°C. All samples were complemented with pBS-SK+ plasmid (Stratagene) to an identical amount (500 ng/well). Similar experiments were performed with a negative control consisting of the empty TK-pGL3-basic plasmid (Promega). After transfection, cells were cultured in DMEM supplemented by 0.2% FBS for 24 hours to strengthen cell membrane before addition of FAs. Afterwards, cells were transactivated for 24 hours in absence or presence of omega-3 FAs in concentrations varying between 1–15 *μ*M to reflect biological plasma or red blood cells concentration of FAs [14]. Cells were treated with either solvent (dimethyl sulfoxide (DMSO), 0.01% final concentration), or treatments of EPA and/or DHA (Sigma-Aldrich, Oakville, ON, Canada). Briefly, pure EPA or DHA was dissolved by serial dilution to 1, 5, 10, and 15 *μ*M in DMSO. For mixtures of 5:5, 15:5, and 5:15 *μ*M EPA:DHA, the dissolved individual omega-3 FAs at appropriate concentrations were mixed together. The luciferase activity was quantified with a luminometer (Bertholus, LB956V) and expressed as fold induction in the presence of variable doses of omega-3 FAs over control. Ciprofibrate (250 *μ*M) (Sigma-Aldrich) was used as a positive control of induction. The assays were performed in triplicates. The experiment was conducted in duplicate.

### 2.3. Data Analysis

Firefly luciferase activities were normalized with the corresponding Renilla luciferase reporter activity as internal control. Fold induction was calculated by taking the control DMSO (Sigma-Aldrich) as baseline.

## 3. Results

**Figure 1:**
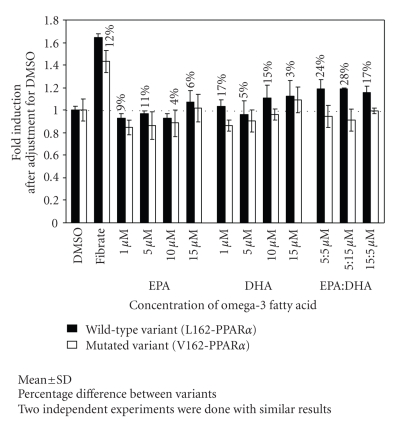
Transcriptional activity by L162-PPAR*α* and V162-PPAR*α* in HepG2 cells supplemented with EPA, DHA, and mixtures of EPA:DHA. The DR1-PPRE-TKpGL3 reporter construct (100 ng) was cotransfected with the pRL-NULL plasmid (30 ng) in HepG2 cells in presence of 10 ng pSG5-hPPAR*α* wild-type (black bars) or mutated (white bars) and pSG5-mRXR*α* (10 ng) plasmids. Cells were subsequently treated or not with ciprofibrate (250 *μ*M) or varying concentrations and mixtures of EPA and/or DHA for 24 hours. Values were normalized to internal Renilla luciferase activity as described in materials and methods and expressed as fold-induction relative to the control (TK-pGL3) set at 1. Values are representative of 2 independent experiments realized in triplicates.

Transient transfection assays in human hepatoma HepG2 cells were done to compare L162-PPAR*α* to V162-PPAR*α* transcriptional activity. In sum, two independent transients' transfection assays were performed with similar results for transcriptional activity. The V162-PPAR*α* variant showed similar basal transcriptional activity after treatment with DMSO compared with L162-PPAR*α* on the DR1x6-PPRE. For positive control, the presence of the PPAR synthetic ligand, ciprofibrate, showed enhanced transactivation activity in V162-PPAR*α* compared with L162-PPAR*α* (Figure 1). Most importantly, the results from this functional study demonstrate that increase in activity in the V162-PPAR*α* variant did not reach the same level of extent of transcriptional activity as the L162-PPAR*α* variant in all replicates and doses of omega-3 FAs.

In more details, the addition of 5 and 15 *μ*M EPA resulted in an increased in activity with respect to basal level of EPA of 1 *μ*M in L162-PPAR*α* variant, yet only 15 *μ*M EPA resulted in a slight increase in transcriptional rate compared to DMSO (Figure 1). In the same way, the addition of 5, 10, and 15 *μ*M EPA resulted in an higher activity, with respect to basal level in V162-PPAR*α* variant (Figure 1). Nevertheless, transcriptional activity by the L162-PPAR*α* variant compared to V162-PPAR*α* variant was 9%, 11%, 4% and 6% consistently greater with 1, 5, 10, and 15 *μ*M of EPA (Figure 1) representing functional differences between the variants.

Similarly, the addition of DHA enhanced transcriptional activity at most concentrations in both the L162-PPAR*α* and V162-PPAR*α* variant compared to basal level of DHA (Figure 1). However, only 10 or 15 *μ*M of DHA in the L162-PPAR*α* increased activity compared to DMSO. Likewise, the addition of 1, 5, 10, or 15 *μ*M of DHA increased to a greater extent the transcriptional activity by the L162-PPAR*α* variant compared to the V162-PPAR*α* variant (17%, 5%, 15%, and 3%; resp.) (Figure 1).

In addition, EPA:DHA mixtures tested showed a marked increase in transcriptional activity that was higher with respect to individual FA transcriptional activity or basal activity (Figure 1). Again in the V162-PPAR*α* variant, the ratios of EPA:DHA increased the receptor activity but to a lesser degree than in L162-PPAR*α* (Figure 1). The disparities in transcriptional activity between the L162-PPAR*α* and V162-PPAR*α* variants were even more important: 24%, 28%, and 17% for 5:5, 5:15 and 15:5 *μ*M EPA:DHA ratios, respectively. Overall, even if the individual FAs show a smaller transcriptional activity by PPAR*α* with a larger standard deviation, this transcriptional activity is consistently lower in the V162-PPAR*α* than L162-PPAR*α*. Further, this information is supported by the results of the mixtures of EPA:DHA, where there is clearly an increased transcriptional activity and this effect is of lesser magnitude in V162-PPAR*α* than L162-PPAR*α*.

## 4. Discussion

The present study represents the first examination of the variation in transcriptional activity after omega-3 FA activation in the L162V polymorphic variant. Overall, the use of natural PPAR*α* agonists, such as omega-3 FAs, may influence the activation of PPAR*α* at higher doses. Nevertheless, differences exist in the rates of transcriptional activity by the V162-PPAR*α* and the L162-PPAR*α* variant of the PPAR*α* L162V polymorphism. In addition, the additive effects of EPA and DHA mixtures on transcription rates may reveal supplementary benefits compared to the individual omega-3 FAs.

The results clearly reveal that the V162-PPAR*α* has lower transcriptional activity than the L162-PPAR*α*. Previous research has demonstrated the impact of PPAR*α* on the clearance of TG-rich lipoproteins in humans after treatment with PPAR*α* agonist, fibrates [15]. The plasma TG lowering effect of fibrates can be duplicated in animal studies [16]. In contrast, plasma TGs are elevated in animals lacking PPAR*α* [17]. This data suggest that PPAR*α* adjusts LPL-dependent TG lypolysis by altering expression of pro- and antilipolytic factors [18]. Thus, the current results demonstrate that individuals carrying a V162-PPAR*α* variant may potentially have elevated TG levels due to lower transcription rate of target genes, such as *LPL*. These in vitro results support the numerous human studies in which the PPAR*α* L162V polymorphism exhibited associations with total cholesterol, LDL-cholesterol, apo B, TG, and high-density lipoproteins (HDL)-cholesterol [4–9]. In general, from the current and previous human studies, the V162 allele appears to be associated with a more harmful lipid profile potentially due to a lower transcription rate of target genes with PPREs.

The improve transactivation of both allelic variants following an omega-3 FA activation reveals the importance of stratifying individuals according to their dietary fat intakes including omega-3 FA to demonstrate the influence of PPAR*α* L162V polymorphism on lipid parameters and other metabolic factors. Since the mutation is located in the DNA binding domain, this single nucleotide polymorphism is thought to have an impact on the receptor's ability to bind DNA [12]. While receptors coregulators (i.e., coactivators and corepressors) generally interact with the ligand binding domain of nuclear receptors [19], we cannot exclude that the L162V amino acid substitution affects the PPAR*α*'s ability to adequately separate from cytoplasmic corepressor, transit to the nuclei and/or recruit coactivators, as it was demonstrated for the V227A variant of this receptor [20]. To the best of our knowledge functional studies have never been performed for the PPAR*α* L162V mutation, and the mechanisms at the basis of differential omega-3 FAs-dependent activation of the wild-type and mutated receptors remain to be elucidated. Yet, it appears that the V162-PPAR*α* has the potential to reach comparable transcription rates as L162-PPAR*α* with higher intakes of individual or mixtures of omega-3 FAs. Therefore, the influence of the L162V polymorphic variant may be more apparent in individuals who consume a lower intake of omega-3 FAs. These results are in accordance with previous human studies [10, 11, 21] which examined the effect of the PPAR*α* L162V polymorphism in relation to diet. These previous researchers determined that a high intake of dietary PUFA can lower TG in carriers of the V162-PPAR*α* allele [10, 11] due to higher n-3 FA intakes that may lead to increased activation of PPAR*α*. Finally, a recent study by Caron-Dorval et al. in 2008 [21] demonstrated that plasma TG levels decreased similarly between a group of 28 young men with or without the L162V polymorphism after an intense omega-3 FA supplementation for 4 weeks. These results confirm that dietary modifications including higher amounts of EPA and DHA, which activate PPARs to a greater level, may be an effective method in reducing metabolic risk in those with high-risk allele, such as V162. However, this point requires further investigation to ascertain a precise nutritional recommendation.

An additional purpose of this study was to determine whether EPA, DHA, and combinations of EPA:DHA have differential roles in transcriptional activity. Most studies regarding the effects of n-3 PUFA on blood lipids were conducted with fish oils that contain a mixture of EPA and DHA [22, 23]. Yet, a number of studies have been conducted with EPA and DHA individually. In vitro [24] and animal [25–28] studies suggest that EPA rather than DHA may be a hypotriglyceridemic agent. However, divergent findings have been reported in human studies [29, 30]. Results from the current study with individual FAs indicate that a higher dose of either EPA or DHA can increase transcriptional rate of target genes. However, our results demonstrated that DHA may have a slightly higher transcriptional activity than EPA. A recent study by Sanderson et al. in 2008 [31] showed that DHA behaved as a highly potent inducer of PPAR*α* dependent gene expression compared to other FAs, although they did not examine the effects of EPA or mixtures of these FAs. On the other hand, investigators who examined the effects of oleic acid, EPA, and DHA on intestinal gene expression in mice identified 19, 46, and 41 genes, respectively, that were activated with these fatty acids versus 74 genes with the PPAR*α* agonist [32]. In addition in the current study, all tested concentrations of combination of EPA and DHA induced slightly higher transcription rates than individual FAs. However, it still remains unclear whether EPA and DHA have similar TG lowering potential. Further studies are needed to determine whether EPA and DHA in combination, as they are found naturally in fish oils, have an additive effects on gene expression rates; hence, potentially reducing TG concentrations to a greater extent than individual FAs.

In conclusion, these results indicate that the V162-PPAR*α* variant has lower transcriptional activity than L162-PPAR*α* variant in response to omega-3 FAs; therefore, clearly demonstrating that a nutrient-gene interaction exists between PPAR*α* L162V polymorphism and omega-3 FAs. Further studies are needed to confirm whether this difference in transcriptional activity by PPAR*α* is translated into differences in gene expression levels of physiological target genes. Overall, the functional understanding of omega-3 FAs in relation to PPAR*α* L162V genotypes may allow more targeted individualized dietary advice to maximising the benefit gained by the individual.
